# Supplementary material for: Site-specialization of human oral Porphyromonas species
Source: bioRxiv. 2026 Jun 3:2026.06.02.729646. Preprint. [Version 1] doi: 10.64898/2026.06.02.729646 (PMC13252396; doi:10.64898/2026.06.02.729646)
Supplement: Supplement 2 [file NIHPP2026.06.02.729646v1-supplement-2.pdf]

bioRxiv preprint doi: https://doi.org/10.64898/2026.06.02.729646; this version posted June 3, 2026. The copyright holder for this preprint (which was not certified by peer review) is the author/funder, who has granted bioRxiv a license to display the preprint in perpetuity. It is made available under a CC-BY 4.0 International license.

Supplementary Materials

Extended Data (text and Figs. E1 to E3)

Figs. S1 to S7

Tables S1 to S27

bioRxiv preprint doi: https://doi.org/10.64898/2026.06.02.729646; this version posted June 3, 2026. The copyright holder for this preprint (which was not certified by peer review) is the author/funder, who has granted bioRxiv a license to display the preprint in perpetuity. It is made available under a CC-BY 4.0 International license.

Extended Data

Phylogeny of *Porphyromonas* taxa

To establish a curated framework for identifying human oral genomes within the genus *Porphyromonas*, we first examined the evolutionary relationships among all 343 quality-filtered publicly available genomes. Many were deposited as *P.* sp., and numerous metagenome-assembled genomes (MAGs) lacked standardized species designations, creating ambiguity that complicated comparative analyses. We resolved these inconsistencies by constructing a phylogeny of the *Porphyromonas* genus from universal bacterial marker genes (Extended Data Fig. E1). To standardize taxonomic assignments, we used the Genome Taxonomy Database Toolkit (GTDB-Tk; release r226) to provide species-level assignments independent of historical nomenclature. The resulting tree revealed well-supported monophyletic clades representing coherent evolutionary groups comprising genomes with recognized species names, Human Microbial Taxon (HMT) designated taxa, or unnamed *P.* sp. genomes. This phylogenetic framework thus provided a foundation for taxonomic harmonization and the identification of candidate human oral genomes.

We next annotated each genome according to its isolation metadata from NCBI and assigned genomes to one of three putative categories: human oral, human non-oral, or animal–as shown in the 'Ecology layer' of Extended Data Fig. E1. Because isolation metadata may reflect sampling bias rather than true ecological habitat, taxa were considered potentially human oral when at least one genomic member had a documented origin from the oral cavity or upper respiratory tract. Using this criterion, human oral taxa comprised: *P. gingivalis*, *P. endodontalis*, *P. catoniae*, *P.* sp. HMT-275, *P.* sp. HMT-278, *P. pasteri*, and several unnamed *P.* sp. clades–labeled according to the closest named species as indicated in the 'Taxa' layer of Extended Fig.

bioRxiv preprint doi: https://doi.org/10.64898/2026.06.02.729646; this version posted June 3, 2026. The copyright holder for this preprint (which was not certified by peer review) is the author/funder, who has granted bioRxiv a license to display the preprint in perpetuity. It is made available under a CC-BY 4.0 International license.

E1. Non-oral human *Porphyromonas* were included in the phylogeny to provide ecological context. Notably, human oral taxa are phylogenetically closer to animal taxa than to *Porphyromonas* isolated from other human body sites. *Porphyromonas asaccharolytica* (HMT-547) and *P. uenonis* (HMT-785) are listed in HOMD (v4.1) with 'uncertain' or 'primarily vaginal' habitats, and along with *P. vaginalis* they group in the same branch. The genome *P.* sp. '31_2' (GCA_000712235.1) deposited as *Porphyromonas* was classified as *Parabacteroides distasonis* and showed relatedness to *Tannerella*. This isolation metadata-based approach generated a set of candidate human oral genomes pending ecological validation.

Ecological validation of named taxa across the human body

To assess whether candidate human oral taxa recruited reads from oral habitats, we performed competitive mapping of shotgun metagenomic sequence data to quality-filtered type strain genomes. We used metagenomic data from the Human Microbiome Project which sampled four human body sites in healthy individuals: the mouth, stool, vagina, and skin. Within these sites, data were available from nine oral subsites, four skin subsites, and three vaginal subsites. We also included data from a study of subgingival plaque in periodontitis (Extended Data Fig. E2). This analysis confirmed that taxa identified as oral candidates based on phylogeny and metadata consistently recruited reads from oral metagenomes.

Porphyromonas uenonis was detected primarily in vaginal samples, but also at low prevalence in oral sites. *Porphyromonas asaccharolytica* and *P. vaginalis* also predominantly recruited reads from vaginal metagenomes, but had negligible recruitment from oral metagenomes. *Porphyromonas bennonis*, *P. somerae*, and *P. miyakawae* were not detected across any surveyed body site. Notably, despite its historical role as the reference species of the genus, *P. asaccharolytica* showed no oral detection, indicating it is unlikely to be a constituent of

bioRxiv preprint doi: https://doi.org/10.64898/2026.06.02.729646; this version posted June 3, 2026. The copyright holder for this preprint (which was not certified by peer review) is the author/funder, who has granted bioRxiv a license to display the preprint in perpetuity. It is made available under a CC-BY 4.0 International license.

the oral microbiome. The detection of *P. uenonis* in both vaginal and some oral samples suggests that some *Porphyromonas* taxa can occupy multiple mucosal niches rather than being restricted to a single body site.

A phylogenetically and ecologically defined oral set

By constructing phylogenomic relationships, resolving taxonomic identity and validating the ecological distribution of named species across body sites, we delineated a human oral subset of genomes for the genus *Porphyromonas*. This set includes both named taxa with established type strains and as yet unnamed clades representing potential novel taxa. This set of 191 genomes forms the curated reference set of genomes for the metapangenomic analyses in the main text.

Pangenome of 191 human oral *Porphyromonas* genomes

Pangenome analysis of all 191 human oral *Porphyromonas* genomes prior to dereplication (Extended Data Fig. E3) confirmed the existence of coherent phylogenetic groups but also revealed within-group structure that dereplication, by design, compresses. *Porphyromonas gingivalis* was represented by 99 genomes before dereplication, reflecting high availability of isolates for this disease-associated taxon, but collapsed to a single representative after dereplication, consistent with its extremely low intraspecies genome diversity. *Porphyromonas pasteri* had only one genome eliminated by dereplication, which corresponded to the *P. bobii* strain 'EMRHCC 6C' which was deposited twice. Overall, the 12 color-coded genomic groups shown in Extended Data Fig. E3 represent 7 named human oral species (see main text Fig. 1) together with their within-group structure (*P. catoniae*, 3 clades; *P.* sp. HMT-275, 2 clades; *P. endodontalis*, 2 clades; and *P. uenonis*, 2 clades).

bioRxiv preprint doi: https://doi.org/10.64898/2026.06.02.729646; this version posted June 3, 2026. The copyright holder for this preprint (which was not certified by peer review) is the author/funder, who has granted bioRxiv a license to display the preprint in perpetuity. It is made available under a CC-BY 4.0 International license.

Multiple strains, including type strains, have been independently sequenced and deposited by different culture collections; these duplicated assemblies are visible in the pre-dereplicated pangenome. For example, *P. endodontalis* ATCC 35406 = NCTC13058 = FDAARGOS 1506 has three independent entries. The inclusion of such duplicates in the pre-dereplicated set enables a quality check on the resulting pangenome. Sequencing of genomes of the same strain multiple times is expected to produce near-identical results, thus serving to measure the accuracy of genomic sequencing. Subtle gene-content differences among duplicates are expected and likely reflect sequencing error rate, assembly workflow, and cultivation passage.

For *Porphyromonas* as a whole, the majority of gene clusters in the pre-dereplicated pangenome carry at least one functional annotation across COG, Pfam, KOfam, or CAZyme databases, indicating that comparative metabolic and functional analyses are well-supported by the available genomic information.

bioRxiv preprint doi: https://doi.org/10.64898/2026.06.02.729646; this version posted June 3, 2026. The copyright holder for this preprint (which was not certified by peer review) is the author/funder, who has granted bioRxiv a license to display the preprint in perpetuity. It is made available under a CC-BY 4.0 International license.

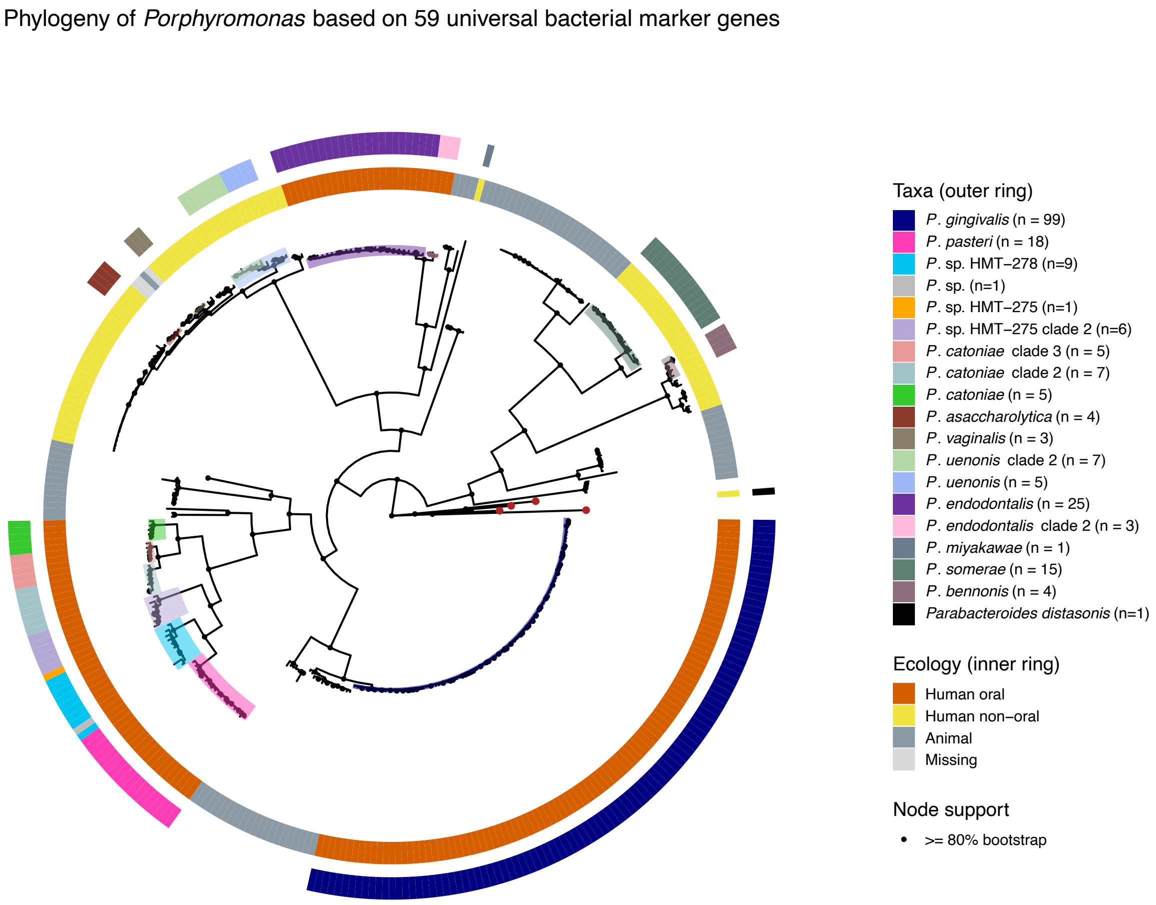

Extended Fig. E1. Phylogenomic framework of the genus *Porphyromonas*. Maximum-likelihood phylogeny of 343 quality-filtered *Porphyromonas* genomes inferred from concatenated amino-acid sequences of 59 conserved bacterial marker genes (Bacteria_71 set). Branch lengths represent amino-acid substitutions per site. Node support was assessed using 1,000 bootstrap replicates (UFBoot); only ≥80% are shown (black dots). The tree is rooted using outgroup genomes from *Bacteroides pyogenes*, *Prevotella melaninogenica*, *Tannerella forsythia*,

bioRxiv preprint doi: https://doi.org/10.64898/2026.06.02.729646; this version posted June 3, 2026. The copyright holder for this preprint (which was not certified by peer review) is the author/funder, who has granted bioRxiv a license to display the preprint in perpetuity. It is made available under a CC-BY 4.0 International license.

and *Tannerella serpentiformis* (shown with red dots at the tips). Metadata annotations are indicated in the two rings. The outer ring denotes names of curated human species-level taxa along with the number of genomes in each taxon. Names are not shown for animal taxa. The inner ring denotes the ecology (host and body site) for curated genomes, classifying them as human oral, human non-oral, animal, or missing isolation metadata.

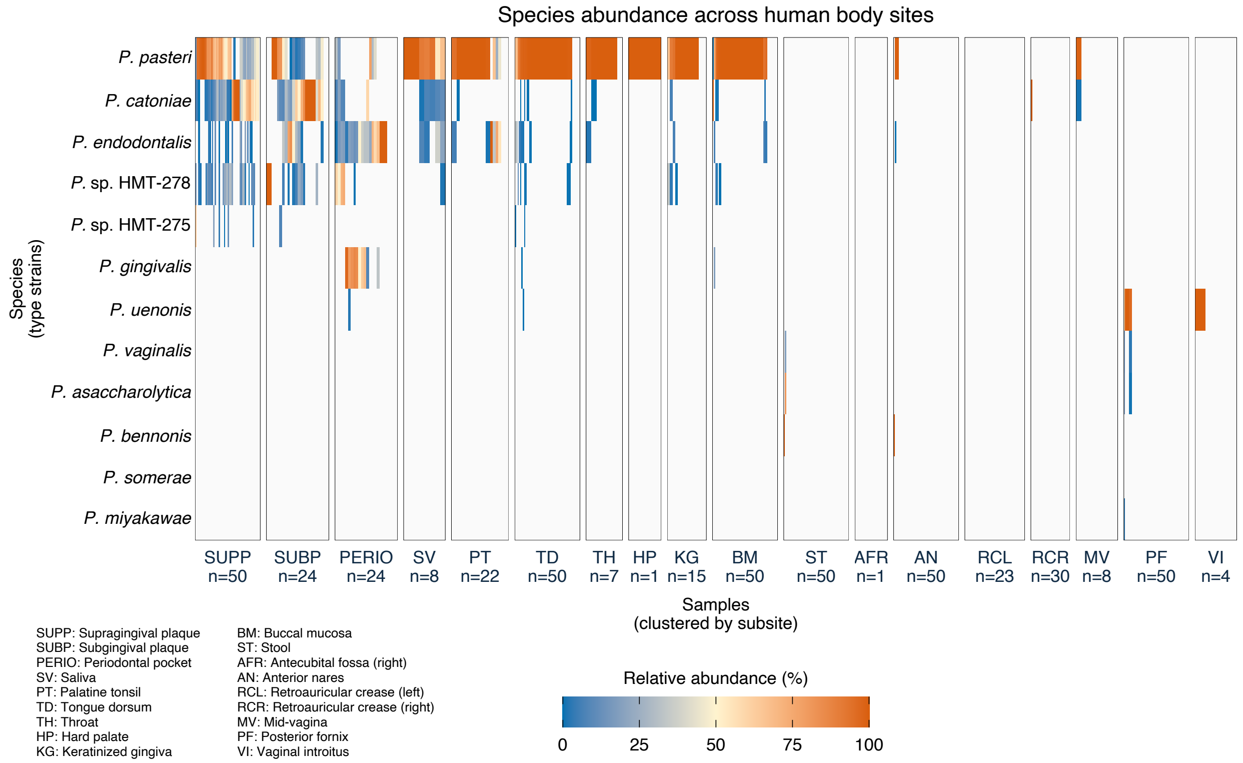

Extended Fig. E2. Distribution of *Porphyromonas* type strains across human body sites. Relative abundance of type strains representing 12 named *Porphyromonas* species, expressed as a fraction of total *Porphyromonas* abundance, following competitive mapping to up to 50 metagenomes per body subsite from the Human Microbiome Project healthy cohort. Relative abundance is expressed as the fraction of the Q2-Q3 mean depth of coverage for each genome within a given sample. Body subsites are ordered left to right as oral (SUPP, SUBP, PERIO, SV,

bioRxiv preprint doi: https://doi.org/10.64898/2026.06.02.729646; this version posted June 3, 2026. The copyright holder for this preprint (which was not certified by peer review) is the author/funder, who has granted bioRxiv a license to display the preprint in perpetuity. It is made available under a CC-BY 4.0 International license.

PT, TD, TH, HP, KG, BM), stool (ST), skin (AFR, AN, RCL, RCR), and vagina (MV, PF, VI). Samples within each subsite are hierarchically clustered by Bray-Curtis dissimilarity with Ward linkage. Light grey indicates no detectable abundance.

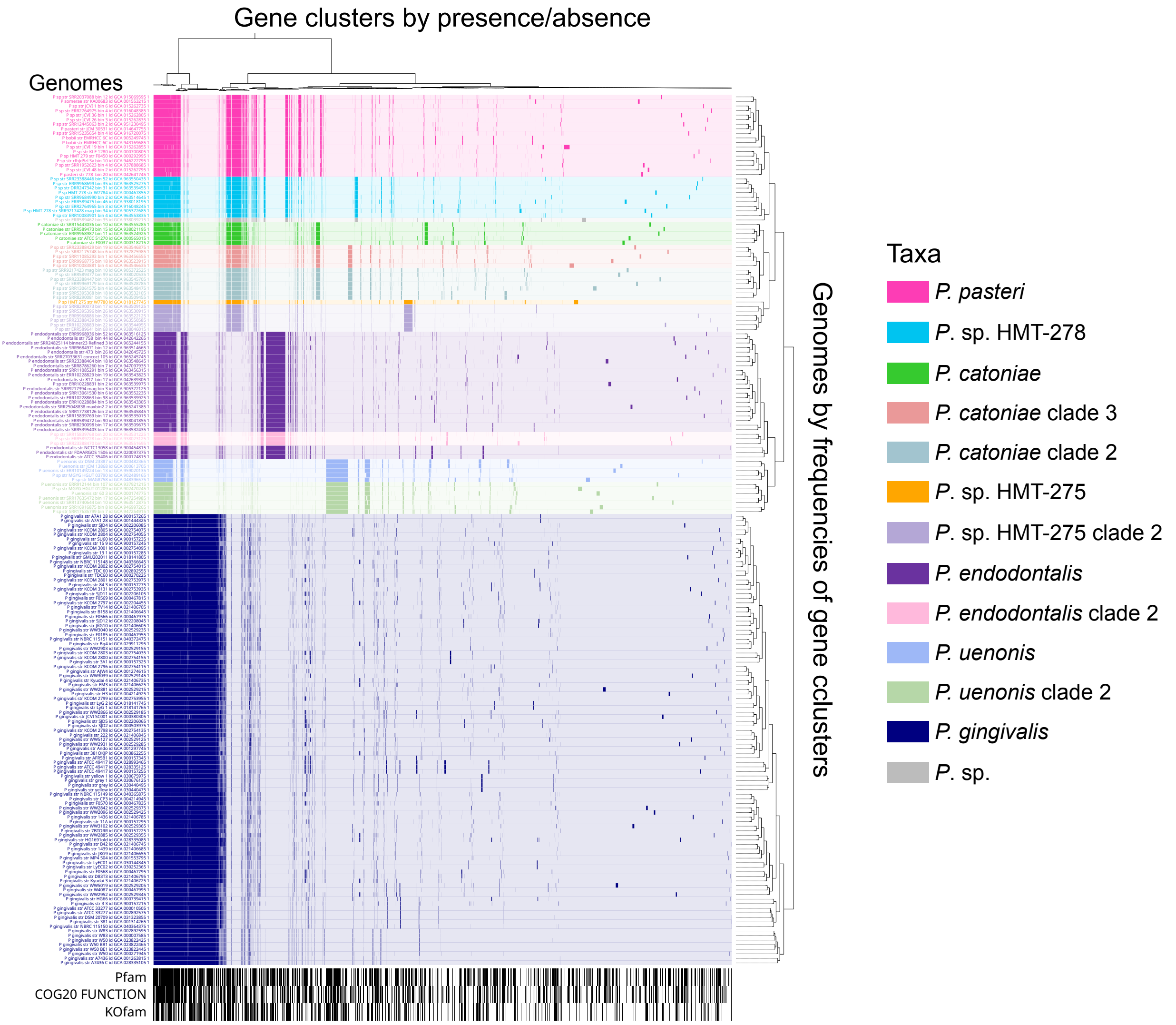

Extended Fig. E3. Pangenome of human oral *Porphyromonas* genomes prior to dereplication (n = 191). All 191 high-quality human oral *Porphyromonas* genomes are organized by gene content. Predicted open-reading frames (ORFs) were translated to amino-acid sequences, compared using BLASTp, and grouped into homologous gene clusters (vertical bars)

bioRxiv preprint doi: https://doi.org/10.64898/2026.06.02.729646; this version posted June 3, 2026. The copyright holder for this preprint (which was not certified by peer review) is the author/funder, who has granted bioRxiv a license to display the preprint in perpetuity. It is made available under a CC-BY 4.0 International license.

via Markov Clustering Algorithm (MCL; inflation =10). Genomes are hierarchically clustered by gene-cluster frequencies (dendrogram to the right) and are color-coded by genomic group. Black bars at the bottom indicate gene clusters with associated functional annotation.

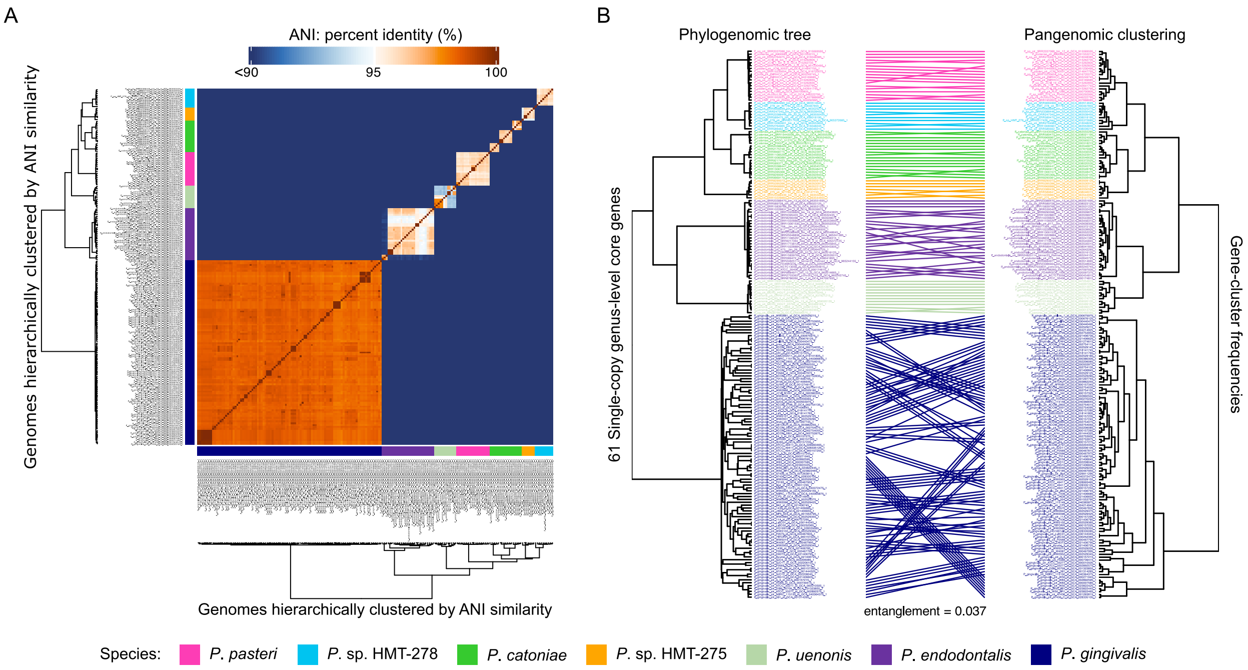

Fig. S1. ANI heatmap and tanglegram for human oral *Porphyromonas* genomes. (A) Pairwise ANI heatmap of 191 high-quality human oral *Porphyromonas* genomes reveals that most species show strong intraspecies similarity (>95%) and sharp interspecies boundaries. Values range from 90% (blue) to 100% (red), with a midpoint 95% (white). Genomes are hierarchically clustered by ANI values. (B) Tanglegram comparing a maximum-likelihood phylogenomic tree (left; 61 single-copy core genes, WAG model, 1,000 bootstraps) and a pangenomic tree (right; derived from gene cluster frequency data). Matching genomes are connected by lines arranged to minimize entanglement; labels and lines are color-coded by genomic group designation.

bioRxiv preprint doi: https://doi.org/10.64898/2026.06.02.729646; this version posted June 3, 2026. The copyright holder for this preprint (which was not certified by peer review) is the author/funder, who has granted bioRxiv a license to display the preprint in perpetuity. It is made available under a CC-BY 4.0 International license.

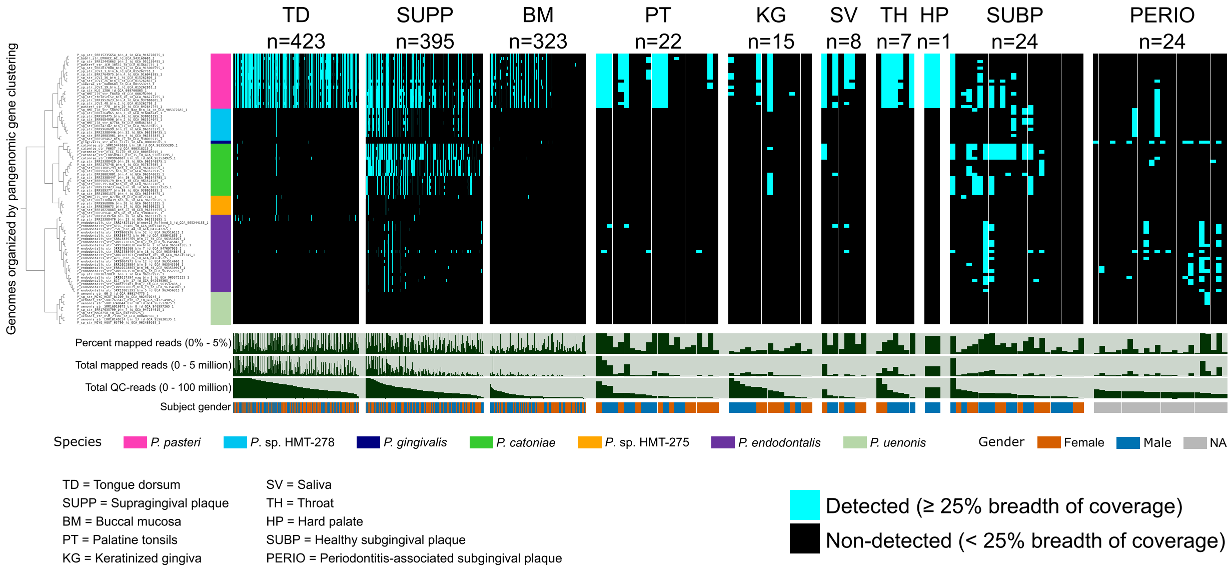

Fig. S2. Binary detection of reference *Porphyromonas* genomes across oral sites. Following breadth of coverage, genomes were considered detected (cyan) in a sample if ≥ 25% of nucleotides had at least one mapped read. Detection is shown as a binary presence/absence plot for 84 reference genomes across the 1,242 human metagenomes from nine healthy oral sites and one periodontitis-associated site. Genomes are ordered by gene cluster frequencies (as in Fig. 1); metagenomes are grouped by site and sorted by decreasing read count.

bioRxiv preprint doi: https://doi.org/10.64898/2026.06.02.729646; this version posted June 3, 2026. The copyright holder for this preprint (which was not certified by peer review) is the author/funder, who has granted bioRxiv a license to display the preprint in perpetuity. It is made available under a CC-BY 4.0 International license.

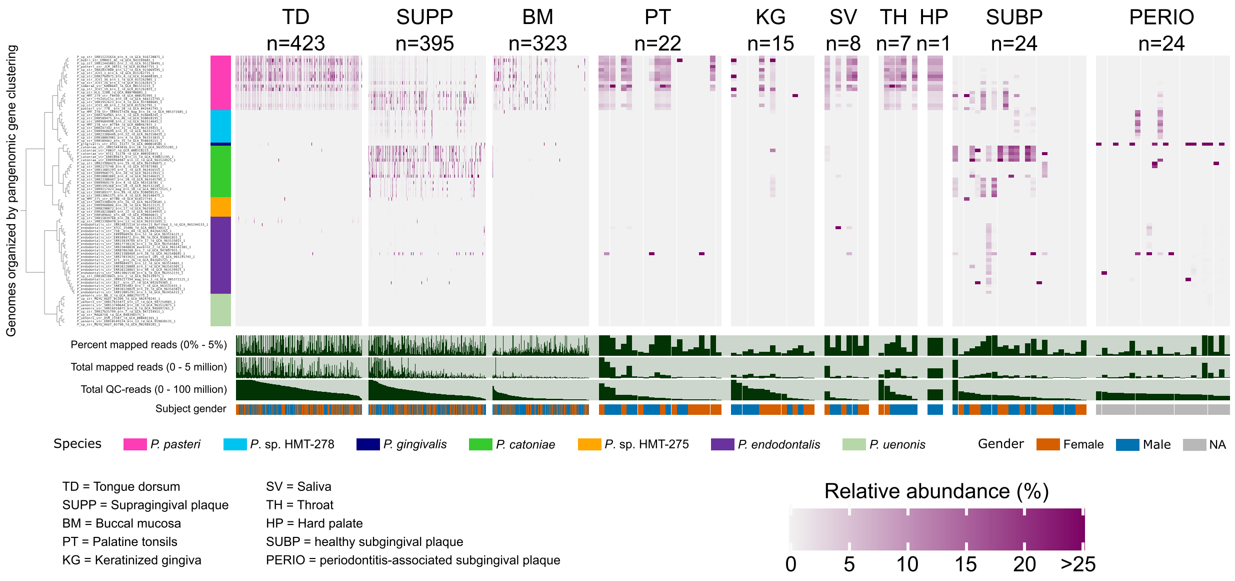

Fig. S3. Genome-level relative abundance heatmap. Relative abundance values range from 0% (black) to 25% (magenta) and were calculated as the genomic Q2-Q3 mean depth of coverage divided by the total Q2-Q3 mean depth of coverage across all *Porphyromonas* genomes in a sample. Genomes and metagenomes are ordered as in Fig. S2.

bioRxiv preprint doi: https://doi.org/10.64898/2026.06.02.729646; this version posted June 3, 2026. The copyright holder for this preprint (which was not certified by peer review) is the author/funder, who has granted bioRxiv a license to display the preprint in perpetuity. It is made available under a CC-BY 4.0 International license.

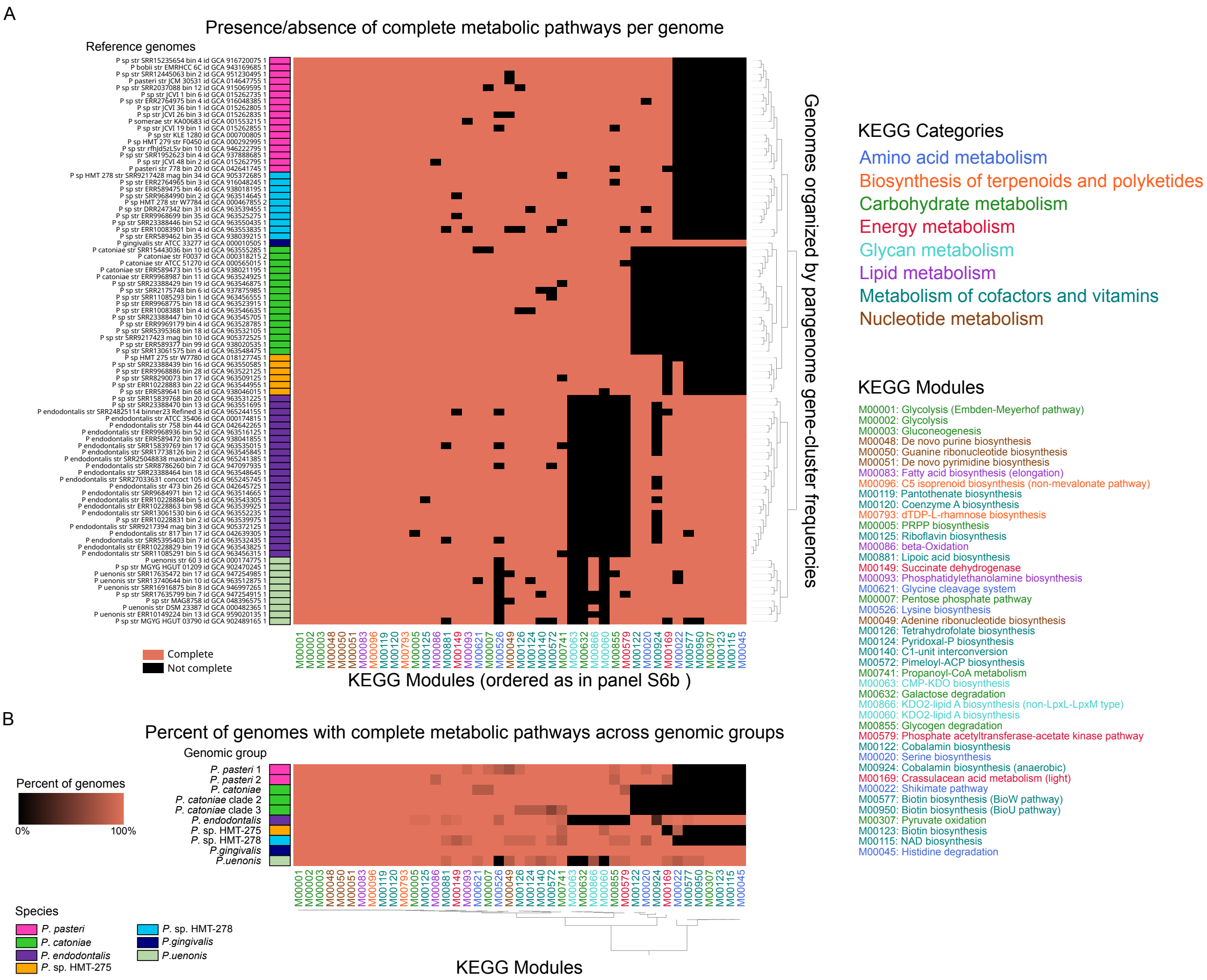

Fig. S4. Metabolic completeness across *Porphyromonas* reference genomes and genomic groups. A) Binary plots showing the presence or absence of complete metabolic pathways in 84 reference genomes. A pathway was considered complete if ≥ 75% of the required KEGG module enzymes were present. B) Heatmap showing the percentage of genomes, within genomic groups, in which each metabolic pathway is complete. Modules are hierarchically clustered by Euclidean distance and Ward linkage. Genomic groups have the same order as in Fig. 3.

bioRxiv preprint doi: https://doi.org/10.64898/2026.06.02.729646; this version posted June 3, 2026. The copyright holder for this preprint (which was not certified by peer review) is the author/funder, who has granted bioRxiv a license to display the preprint in perpetuity. It is made available under a CC-BY 4.0 International license.

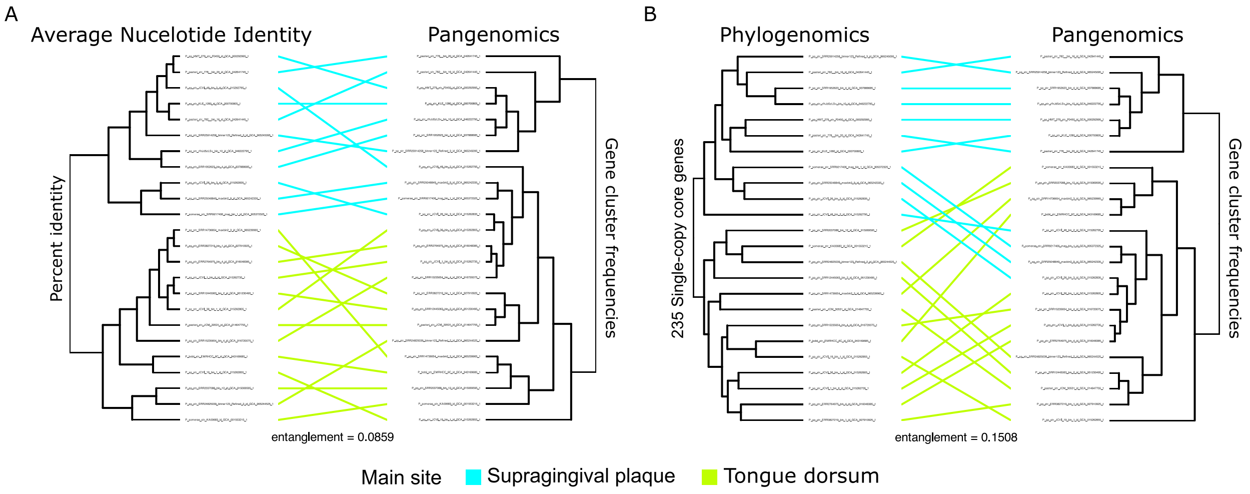

Fig. S5. Comparison of *P. pasteri* ecotypes clustering by phylogeny, pangenome, and ANI. Two tanglegrams compare genome clustering based on (A) ANI vs pangenomic analyses, (B) phylogenomic versus pangenomic analyses. Genome labels and connecting lines are color-coded by the primary oral site: tongue dorsum or supragingival plaque. ANI and phylogenomic trees are from analyses presented in Fig. 4. A *P. pasteri* pangenome was constructed to generate a gene-cluster frequencies dendrogram.

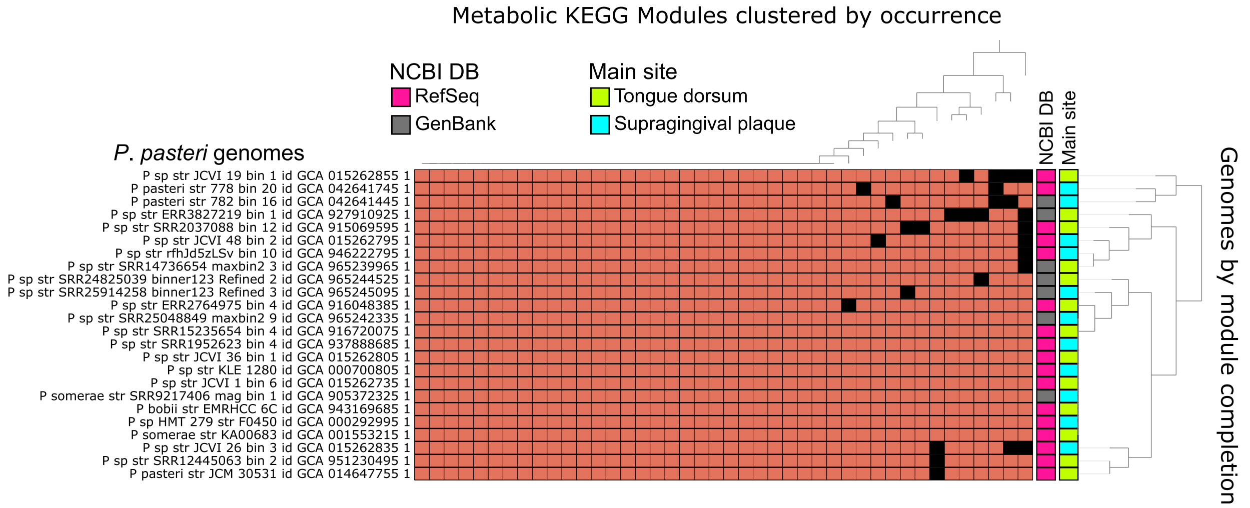

Fig. S6. Metabolic capacity of *P. pasteri* ecotypes. Binary plot showing the presence or absence of complete metabolic pathways (≥75% of required KEGG module enzymes). Across *P. pasteri*

bioRxiv preprint doi: https://doi.org/10.64898/2026.06.02.729646; this version posted June 3, 2026. The copyright holder for this preprint (which was not certified by peer review) is the author/funder, who has granted bioRxiv a license to display the preprint in perpetuity. It is made available under a CC-BY 4.0 International license.

genomes with distinct site preference: tongue dorsum or supragingival plaque. Labels indicate site preference and NCBI database source (RefSeq or GenBank). Clustering of genomes by module, using Euclidean distances and Ward as linkage, showed limited differences among the specialists.

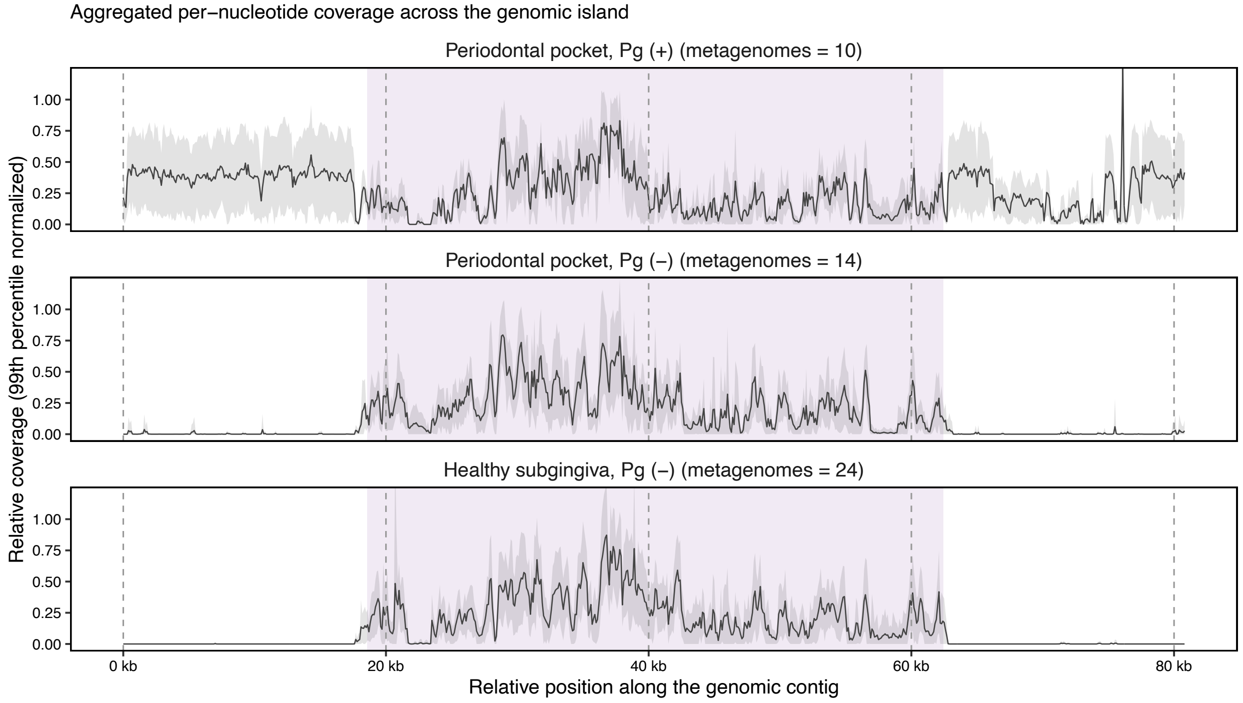

Fig. S7. Metagenomic coverage distribution across a 44 kb mobile conjugative element of *P. gingivalis*. Per-nucleotide coverage of an 80 kb genomic region containing a mobile conjugative element in *P. gingivalis* ATCC 33277 across subgingival plaque metagenomes shows significant coverage despite the absence of the bacterial chromosome. Per-nucleotide coverage for each sample was normalized to the 99th percentile. Normalized coverage was then aggregated across samples within three groups: periodontitis-associated with *P. gingivalis* detected (metagenomes = 10), periodontitis-associated with *P. gingivalis* not detected (metagenomes = 14), and healthy subgingiva (metagenomes = 24). The group mean is shown as a black line, and the shaded ribbon

bioRxiv preprint doi: https://doi.org/10.64898/2026.06.02.729646; this version posted June 3, 2026. The copyright holder for this preprint (which was not certified by peer review) is the author/funder, who has granted bioRxiv a license to display the preprint in perpetuity. It is made available under a CC-BY 4.0 International license.

indicates ± 1 standard deviation across metagenomes. A purple transparent box indicates the relative position of the mobile conjugative element.

Table S1. Collection of 377 RefSeq *Porphyromonas* genomes. Two-column table listing the Assembly Accession numbers and corresponding internal genome identifiers for RefSeq *Porphyromonas* genomes available in NCBI (n = 377; date of download: June 23, 2025).

Table S2. Evaluation of completeness and contamination of the 377 *Porphyromonas* genomes using CheckM. Columns A-N contain the CheckM (v1.2.3) quality report for each genome. Column O indicates whether each genome passed or failed the quality thresholds of ≥ 90% completeness and < 5% contamination.

Table S3. Evaluation of completeness and contamination of the 377 *Porphyromonas* genomes using CheckM2. Columns A-O contain the CheckM2 (v1.1.0) quality report for each genome. Column P indicates whether each genome passed or failed the quality thresholds of ≥ 90% completeness and < 5% contamination.

Table S4. Assessment of completion and redundancy of marker genes for the 377 *Porphyromonas* genomes. Columns A-F contain anvi-estimate-genome-completion report of universal bacterial marker genes (Bacteria_71 set). Column G indicates whether genomes passed or failed completion ≥70% and redundancy <10%.

Table S5. Universal bacterial marker genes in the 343 quality-controlled *Porphyromonas* genome collection for phylogenomic analysis. The table presents the 71 genes from the Bacteria_71 universal bacterial marker set, indicating the ones selected for phylogeny analysis. Column A shows the gene names, and Column B indicates whether each gene was identified in 97% of the 343 quality-controlled *Porphyromonas* and outgroup genomes and was therefore

bioRxiv preprint doi: https://doi.org/10.64898/2026.06.02.729646; this version posted June 3, 2026. The copyright holder for this preprint (which was not certified by peer review) is the author/funder, who has granted bioRxiv a license to display the preprint in perpetuity. It is made available under a CC-BY 4.0 International license.

included in phylogenomic tree construction. Of the 71 genes, 59 met this criterion and were retained for phylogeny.

Table S6. GTDB classification of 343 quality-controlled *Porphyromonas* genomes.

Taxonomic assignments for the 343 quality-controlled *Porphyromonas* genomes based on the GTDB-Tk classify_wf workflow (v2.4.1, GTDB release r226). Columns include our internal genome identifiers and GTDB taxonomy from domain to species.

Table S7. Metadata for 343 quality-controlled *Porphyromonas* genomes. Columns list Genome ID, Ecological Source (human oral, human non-oral, or non-human oral taxon), Assigned Species, NCBI Organism Name, HOMD ID, GTDB taxonomy (Family, Genus, Species), host and isolation details (Host, Isolation Source, Type Material), and assembly information (RefSeq Category, Assembly Level, Sequencing Technology, Assembly Submitter). Genomes ordered by Ecological Source and taxonomy to highlight human oral taxa.

Table S8. Type strains for human *Porphyromonas* species. Selected *Porphyromonas* type strains for competitive mapping across human oral, stool, skin, and vaginal metagenomes from each human. Column A-C: species. Strain ID, Assembly Accession. Column D: Primary body site after mapping.

Table S9. Dereplication clusters and representative genomes of 191 human oral *Porphyromonas* genomes. The table lists 84 dereplication clusters from 191 quality-filtered *Porphyromonas* genomes at 98% ANI using a simple greedy algorithm. Columns include Cluster ID (1 to 84), Cluster Size (number of genomes), Representative Genome (used for competitive mapping), and Member Genomes (all genomes in the cluster). Representative genomes were selected preferentially from isolates over metagenome-assembled genomes (MAGs), based on

bioRxiv preprint doi: https://doi.org/10.64898/2026.06.02.729646; this version posted June 3, 2026. The copyright holder for this preprint (which was not certified by peer review) is the author/funder, who has granted bioRxiv a license to display the preprint in perpetuity. It is made available under a CC-BY 4.0 International license.

type or reference strain status, genome quality (e.g., contiguity), presence in HOMD, and host/isolation source. Clusters are ordered by decreasing cluster size.

Table S10. Pairwise ANI among 191 high-quality human oral *Porphyromonas* genomes. Pairwise average nucleotide identity (ANI) values for 191 high-quality *Porphyromonas* genomes calculated using pyANI with the ANIb method are presented. Rows and columns are labeled by Genome ID, with identity values ranging from 0.00 to 1.00. This matrix was used to validate pangenomic groupings and to guide dereplication at 98% ANI. Corresponding ANI values are visualized as a heatmap in Fig. S1.

Table S11. Summary mapping metadata for 1,242 oral metagenomes. Metadata includes the oral site, total quality-filtered reads, total mapped reads, fraction of mapped reads, and subject gender.

Table S12. Breadth of coverage for representative dereplicated *Porphyromonas* genomes across 1,242 oral metagenomes. The table shows the breadth of coverage for each of the representative dereplicated genomes (rows, labeled by Genome ID) across 1,242 metagenomes from 9 healthy oral sites and 1 periodontitis-associated site (subgingival plaque). Breadth of coverage is defined as the fraction of nucleotides in a genome covered at least 1x, with values ranging from 0.00 to 1.00. Mapping was performed competitively against the dereplicated reference set, and coverage metrics were extracted from the resulting profiles.

Table S13. Genome-level relative abundance across 1,242 oral metagenomes. This table reports the relative abundance of each representative dereplicated genome across 1,242 oral metagenomes from 9 healthy and one disease-associated site. Genome-level relative abundance was calculated as the Q2-Q3 mean depth of coverage of a genome divided by the sum of Q2-Q3

bioRxiv preprint doi: https://doi.org/10.64898/2026.06.02.729646; this version posted June 3, 2026. The copyright holder for this preprint (which was not certified by peer review) is the author/funder, who has granted bioRxiv a license to display the preprint in perpetuity. It is made available under a CC-BY 4.0 International license.

depths of coverage across all genomes detected in the same sample. Rows correspond to genomes and columns to metagenomes.

Table S14. Relative abundance of *Porphyromonas* genomic groups across oral sites. The table contains the aggregated genome relative abundance into 10 genomic groups across the 1,242 oral metagenomes. Relative abundance for each genomic group was calculated as the sum of the relative abundance of its constituent genomes (data from Table S13). Rows correspond to genomic groups, and columns to oral metagenomes. Values represent the fraction of total genomic abundance contributed by each group in a sample, ranging from 0.0% to 100.0%.

Table S15. Oral site prevalence of *Porphyromonas* genomic groups. The table reports the percent of samples in which each *Porphyromonas* genomic group is detected across 9 healthy-associated and one disease-associated oral site. A genomic group was considered present in a sample if at least one of its constituent genomes reached a breadth of coverage ≥25% (data from Table S12). Rows correspond to taxa and columns to oral sites. Values are expressed as a percentage of samples in which each genomic group is detected per site, with the total number of samples per site shown in the column headers. To ease comparison with the relative abundance from Fig. 4, genomic groups and sites are in the same order.

Table S16. Pairwise statistics for *Porphyromonas* genomic groups across oral sites. The table reports contingency-associated metrics, abundance correlations, and dominance comparisons for pairs of genomic groups. Rows correspond to taxon pairs within a site. Columns include sample counts for each detection category, odds ratios, and Fisher's exact test statistics, Spearman rank correlations among informative samples, and centered log-ratio (CLR) dominance comparisons for co-detected pairs. False discovery rate (FDR) corrected p-values are provided for multiple-testing adjustment.

bioRxiv preprint doi: https://doi.org/10.64898/2026.06.02.729646; this version posted June 3, 2026. The copyright holder for this preprint (which was not certified by peer review) is the author/funder, who has granted bioRxiv a license to display the preprint in perpetuity. It is made available under a CC-BY 4.0 International license.

Table S17. Completeness of metabolic modules across 84 reference *Porphyromonas* genomes. Percent of completeness of metabolic modules for each of the 84 reference genomes for modules where at least 75% of the necessary enzymes to complete the pathway are present. 43 of 188 modules are complete. Columns A and B include module ID and description; columns C-CH show percentages ranging from 75% to 100%.

Table S18. Quality assessment of non-RefSeq *Porphyromonas pasteri* genomes. The table summarizes genome quality metrics for 21 non-RefSeq *P. pasteri* genomes. Columns include GTDB Classification (v2.4.1; r226), Bacteria_71 Completion (≥70%), Bacteria_71 Redundancy (<10%), CheckM v1.2.3 Completeness (≥90%), CheckM v1.2.3 Contamination (<5%), CheckM2 v1.1.0 Completeness (≥90%), CheckM2 v1.1.0 Contamination (<5%), and Quality Status (Passed/Not Passed), indicating whether a genome met all thresholds. Seven out of 21 genomes passed all quality criteria.

Table S19. Dereplication of RefSeq and non-RefSeq *Porphyromonas pasteri* genomes for site preference validation. The table summarizes dereplication of all *P. pasteri* genomes used for taxon-specific competitive mapping. Dereplication was performed at 98% ANI across RefSeq and non-RefSeq genomes. Columns include Cluster ID, Cluster Size, Representative Genome, Genomes in Cluster, and NCBI Category (RefSeq/non-RefSeq). The seven non-RefSeq genomes formed their own clusters. Dereplication resulted in 24 *P. pasteri* clusters (7 non-RefSeq and 17 RefSeq).

Table S20. Pfam functional enrichment report in *Porphyromonas pasteri* genomes specialized to the tongue dorsum or supragingival plaque. Results of Pfam functional enrichment analysis in *P. pasteri* genomes are primarily distributed in tongue dorsum (TD) or supragingival plaque (SUPP). Columns include the source (Pfam), Enrichment Score,

bioRxiv preprint doi: https://doi.org/10.64898/2026.06.02.729646; this version posted June 3, 2026. The copyright holder for this preprint (which was not certified by peer review) is the author/funder, who has granted bioRxiv a license to display the preprint in perpetuity. It is made available under a CC-BY 4.0 International license.

Unadjusted p-value, Adjusted q-value, Associated Groups (TD, SUPP, NA), Function Accession ID, Associated Gene Cluster IDs, counts of genomes with the function in each group (p_SUPP_clade, p_TD_clade, N_SUPP_clade, N_TD_clade), and validation flags (q-value <0.05, present in ≥50% of enriched group, present in <50% of non-enriched group, enriched with high ratio). Only one Pfam function was significantly enriched. See Methods "Functional enrichment analysis" for details.

Table S21. COG20 functional enrichment report in *Porphyromonas pasteri* genomes specialized to the tongue dorsum or supragingival plaque. COG20 functional enrichment results for *P. pasteri* gene clusters from TD and SUPP. Columns are as in Table S20. Only one COG20 **function was significantly enriched.**

Table S22. KOfam functional enrichment report in *Porphyromonas pasteri* genomes associated with tongue dorsum or supragingival plaque. KOfam functional enrichment results for *P. pasteri* gene clusters from TD and SUPP. Columns are as in Table S20. No functions were significantly enriched.

Table S23. CAZyme functional enrichment report in *Porphyromonas pasteri* genomes specialized to the tongue dorsum or supragingival plaque. CAZyme functional enrichment results for *P. pasteri* gene clusters from TD and SUPP. Columns are as in Table S20. No functions were significantly enriched.

Table S24. KEGG Module functional enrichment report in *Porphyromonas pasteri* genomes specialized to the tongue dorsum or supragingival plaque. KEGG Module functional enrichment results for *P. pasteri* gene clusters from tongue dorsum (TD) and supragingival plaque (SUPP). Columns are as in Table S20. No KEGG MODULES were significantly enriched.

bioRxiv preprint doi: https://doi.org/10.64898/2026.06.02.729646; this version posted June 3, 2026. The copyright holder for this preprint (which was not certified by peer review) is the author/funder, who has granted bioRxiv a license to display the preprint in perpetuity. It is made available under a CC-BY 4.0 International license.

Table S25. Annotation of *P. gingivalis* mobile genomic island. Gene call coordinates and functional annotations for 45 genes identified as part of a conjugative element in *P. gingivalis* 'ATCC 33277' along with flanking genes for reference. Annotations included: Pfam, NCBI COG20 (category, function, pathway), KEGG (KOfam, Module, BRITE, Class), CAZyme.

Table S26. Nucleotide-level BLAST results for the mobile genomic island and flanking genes. The table contains the top 3 BLASTn results for each gene using HOMD genomes as reference, ranked by identity (%), query coverage (%), and lowest e-value (columns C-F). Taxonomic information is provided for family, genus, and species level (column G-J). Human Microbe Taxon identifier is shown in column J.

Table S27. Protein-level BLAST results for the mobile genomic island and flanking genes. Genes were translated into amino acid sequences, and a protein-protein blast was performed. The table contains the top 3 BLASTp results for each gene using all annotated proteins in HOMD as reference, ranked by identity (%), query coverage (%), and lowest e-value (columns C-F). Taxonomic information is provided for family, genus, and species level (column G-J). Human Microbe Taxon identifier is shown in column J.
